# A novel trial methodology to test interventions with very large effect sizes: the case of dostarlimab in mismatch repair-deficient, locally advanced rectal cancer

**DOI:** 10.1186/s13063-022-06988-1

**Published:** 2022-12-24

**Authors:** Kerrington Powell, Timothée Olivier, Vinay Prasad

**Affiliations:** 1 School of Medicine, Texas A&M Health Science Center, Bryan, TX 77807 USA; 2 Department of Oncology, Geneva University Hospital, 4 Gabrielle-Perret-Gentil Street, 1205 Geneva, Switzerland; 3 Department of Epidemiology and Biostatistics, University of California San Francisco, 550 16th St, 2nd Fl, San Francisco, CA 94158 USA

## Abstract

Dostarlimab (Jemperli, GlaxoSmithKline) is an anti-programmed death receptor-1 monoclonal antibody (anti-PD-1) recently tested in a non-randomized, phase II trial (NCT04165772) which included patients with mismatch repair-deficient, locally advanced rectal cancer. Among the first 12 patients treated with dostarlimab, 100% achieved a clinical complete response with no patients experiencing progression or recurrence to date. Most impressive, none required chemotherapy, radiotherapy or surgery the prevailing standard of care. In this paper, we discuss the impressive results of this trial and how they relate to cancer policy, as well as propose a novel trial methodology to assess dostarlimab.

## Manuscript

Dostarlimab (Jemperli, GlaxoSmithKline) is an anti-programmed death receptor-1 monoclonal antibody (anti-PD-1) recently tested in a non-randomized, phase II trial (NCT04165772) which included patients with mismatch repair-deficient, locally advanced rectal cancer [1]. Among the first 12 patients treated with dostarlimab, 100% achieved a clinical complete response (CR) by all conventional metrics. To date, no patients have experienced progression or recurrence, and, per the protocol, they have not undergone surgery nor chemoradiotherapy, which is considered the standard of care for this condition [1].

Although small, these results are impressive and contribute to a broader, ongoing debate in medicine: under what circumstances should therapies with groundbreaking results be tested in randomized controlled trials (RCTs), or, alternatively, when might results be so impressive that randomization is unnecessary? We explore this question through the lens of dostarlimab.

## The parachute analogy in biomedicine

RCTs are considered the gold standard of medical evidence, gaining popularity in the mid to late twentieth century due to their ability to minimize confounding, solve problems related to time zero (e.g., guarantee time), and limit multiple hypothesis testing [2, 3]. RCTs are particularly useful in biomedicine, where the size of treatment effects are often modest, and interventions are typically delivered at the individual level (with limited clustering or spillover effects) and with the expectation that they will benefit the participant. When non-randomized study designs are used, the observed effect may reflect bias and not the treatment’s true effect size.

While vital for separating small effects from noise or bias, randomized trials may be less important for very large treatment effects. Consider the case of the parachute — an intervention with a 99.99% absolute risk reduction [ARR] in all-cause mortality over a 5- to 15-min time frame [4]. It would appear unwise to randomize individuals to a parachute, as its effect size is so large, it is plainly visible. This point was originally made in 2003 in the *British Medical Journal*. There, Smith and Pell satirically analogized the parachute with biomedical practices by conducting a systematic review of RCTs on parachutes, of which there were none, subtly conveying to proponents of evidence-based medicine that randomized trials should not be conducted for medical practices with obvious benefit [5]. Since its publication, the paper has accrued over 1000 citations.

Most citing articles however misuse the analogy. A citation analysis analyzed 822 articles referencing the original work found that 35 (4.3%) compared a specific medical practice to a parachute, with 22 (out of 35, 63%) of the practices involved already tested in RCTs, and only 6 positive results [6]. The mere testing of a practice in an RCT suggests that academics did not consider it a parachute. Moreover, the ARR in those studies (when available) ranged from 11.0% to 30.8%, far beneath the absolute risk of parachutes.

Second, even medical treatments with large effect sizes pale in comparison to parachutes. Pereira and colleagues examined over 80,000 medical practices in the Cochrane Database, just one of which had a large effect on mortality (− 0.40 risk difference; confidence interval [− 0.59 to − 0.21]) [7]. This is not intended to diminish the accomplishments of medicine but rather to highlight the simple fact that massive effects are rare.

## A brief history: imatinib, the “magic bullet”

At first glance, the efficacy of dostarlimab bares resemblance to that of imatinib (Gleevec, Novartis). In 2001, imatinib changed the landscape of cancer care when 53 out of 54 patients (98%) with chronic myeloid leukemia (CML) achieved a complete hematologic response in a phase I trial [8]. Even after one of the most spectacular early phase trial results in medical science, imatinib was randomized in large, phase III trials versus the standard of care at the time, interferon-alpha plus cytarabine [9]. Imatinib was seen as transformative, narrowing the gap between a CML patient’s and a normal person’s life expectancy, but its status never succumbed to the parachute analogy, instead undergoing rigorous clinical trial testing [10].

## Dostarlimab

The phase II trial results testing dostarlimab presented remarkable results. In addition to a lofty CR rate, dostarlimab is much less toxic and morbid than the current standard of therapy. However, a question arises: does dostarlimab require additional research using RCTs, or is it a parachute? To assess this question, we propose a novel study design. This novel trial design treats a promising intervention as a parachute — initially assigning patients to it without a standard of care control arm — but has a clear, predefined trigger rule to institute randomization. It also relies on one fact: that dostarlimab is a next-in-class molecule, whose parent drugs may have similar properties.

## “Is it a parachute?” trial

Our proposal to assess dostarlimab is the following: patients would be randomized to one of three arms: dostarlimab, nivolumab, or pembrolizumab (anti-PD-1 monoclonal antibodies).

**Fig. 1:**
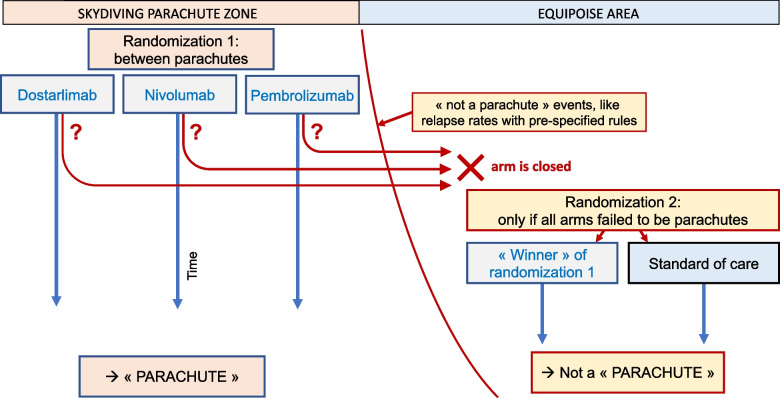
Pragmatic “Is it a parachute?” randomized controlled trial design. Schematic of a unique, pragmatic, randomized controlled trial to evaluate if dostarlimab or other anti-PD-1 drugs are a parachute. Other “parachutes” could be nivolumab and pembrolizumab, with initial randomization between three anti-PD-1 monoclonal antibodies. If “not a parachute” events occur, like relapses with pre-specified rules (e.g., 5%, 10%, 15%), then randomization against the usual standard of care begins

Theoretically, this phase of trial accrual may continue as long as complete responses persist without relapse; nevertheless, if a single arm presents signs of not being a parachute, for instance relapse based on pre-specified stopping rule (i.e., exceeds 5%, 10% or 15%), the arm will be closed. If a second arm also exceeds the stopping rule, it will be closed. However, if all three arms exceeded the stopping rule, then another randomization phase will be triggered. Enrollment will then continue randomizing patients to surgery and chemoradiotherapy — the existing standard of care — vs the winner of the early portion of the trial. A schematic of the trial is depicted in Fig. 1.

This trial design has three advantages. First, it enables researchers to determine if the impressive findings of dostarlimab are a class effect of anti-PD-1 monoclonal antibodies or whether dostarlimab’s mechanism of action in this patient population is intrinsically unique. With such design, one takes the advantage of randomization to answer this question with a three-arm trial within the “parachute zone” (Fig. 1).

Second, this trial is designed for scalability, encouraging a multicenter experiment that will provide data with external validity. The benefits of receiving such strong data will ensure sound clinical and regulatory decision making.

Third, this pragmatic design allows for continuous accrual while in the parachute zone (i.e., until the threshold of evidence supporting one of the three therapies exceeds that of the others). If no strategy turns out to be a parachute (based on pre-specified rules), you have demonstrated that equipoise still exists (“equipoise area” in Fig. 1), justifying further randomization against surgery and chemoradiotherapy as an additional trial arm. The beauty of the trials design is that it pre-specifies under what conditions no patient will be asked to receive the historic, standard of care therapy, and allows for a tremendously successful novel drug to prove its value. Simultaneously, if future results are less than hoped for, randomization of novel to standard is triggered.

## Conclusion

Efficacy results of dostarlimab in patients with mismatch repair-deficient, locally advanced rectal cancer are impressive and hence promising. As a general rule, we caution against premature claims of the drug’s parachute status and, as a consequence, exemption from investigation in RCTs. With further evidence, it is feasible that dostarlimab may rival, or possibly replace, surgery and chemoradiotherapy, especially when considering rates of toxicity and morbidity. The theoretical trial we proposed reconciles the parachute analogy with the need of randomization to answer a critical question. It is in the best interest of our patients that trialists and oncology experts alike view the results of the recent trial evaluating dostarlimab with enthusiasm, tempered by the need for a higher level of evidence. We believe our novel design balances these tensions.

## Data Availability

n/a.
